# Radiation dose is associated with improved local control for large, but not small, hepatocellular carcinomas

**DOI:** 10.1186/s13014-023-02318-0

**Published:** 2023-08-11

**Authors:** Uri Amit, Jahan J Mohiuddin, Andrzej P Wojcieszynski, Joanna Harton, Graeme Williams, Shwetha Manjunath, Nikhil Grandhi, Abigail Doucette, John P Plastaras, James M Metz, Edgar Ben-Josef

**Affiliations:** 1 Department of Radiation Oncology, Perelman School of Medicine, Philadelphia, PA USA; 2 Department of Radiation Oncology, Tel Aviv Medical Center, Tel Aviv, Israel; 3 Levine Cancer Institute, Atrium Health, Charlotte, NC USA; 4 Southeast Radiation Oncology Group, Charlotte, NC USA; 5 Colorado Permanente Medical Group, Denver, CO USA; 6 Genesis Research, Hoboken, NJ USA; 7 Department of Medicine, Washington University School of Medicine, St. Louis, MO USA; 8 Abramson Cancer Center, Perelman School of Medicine, Philadelphia, PA USA

**Keywords:** Radiation, Hepatocellular carcinoma, Radiation-induced liver toxicity

## Abstract

**Background:**

With advances in understanding liver tolerance, conformal techniques, image guidance, and motion management, dose-escalated radiotherapy has become a potential treatment for inoperable hepatocellular carcinoma (HCC). We aimed to evaluate the possible impact of biologically effective dose (BED) on local control and toxicity among patients with HCC.

**Methods and materials:**

Patients treated at our institution from 2009 to 2018 were included in this retrospective analysis if they received definitive-intent radiotherapy with a nominal BED of at least 60 Gy. Patients were stratified into small and large tumors using a cutoff of 5 cm, based on our clinical practice. Toxicity was assessed using ALBI scores and rates of clinical liver function deterioration.

**Results:**

One hundred and twenty-eight patients were included, with a mean follow-up of 16 months. The majority of patients (90.5%) had a good performance status (ECOG 0–1), with Child-Pugh A (66.4%) and ALBI Grade 2 liver function at baseline (55.4%). Twenty (15.6%) patients had a local recurrence in the irradiated field during the follow-up period. Univariate and multivariate Cox proportional hazard analyses showed that only BED significantly predicted local tumor recurrence. Higher BED was associated with improved local control in tumors with equivalent diameters over 5 cm but not in smaller tumors. There was no difference in liver toxicity between the low and high-dose groups.

**Conclusions:**

Higher radiotherapy dose is associated with improved local control in large tumors but not in tumors smaller than 5 cm in diameter. High-dose radiotherapy was not associated with increased liver toxicity.

**Supplementary Information:**

The online version contains supplementary material available at 10.1186/s13014-023-02318-0.

## Background

The evolution of conformal radiotherapy techniques brought enthusiasm for dose escalation to improve oncologic outcomes. Liver neoplasms, once a challenging disease site for external beam radiotherapy, have represented a promising test case of dose escalation in this new therapeutic era. With HCC as the fourth-leading cause of cancer death worldwide and its incidence rising, questions related to the efficacy of therapy are increasingly critical [1].

External beam radiotherapy for HCC is typically reserved for patients with advanced HCC who are poor candidates for a liver transplant or partial hepatectomy [2]. Radiation oncologists now utilize highly modulated beams and image guidance to deliver high-dose radiation to liver tumors with moderate- or ultra-hypofractionation. Treating with higher doses could be especially important in larger tumors, which are more prone to local recurrence after radiation [3, 4]. However, the optimal dose of radiotherapy remains controversial. Some studies have shown that treating HCC with high BED improves outcomes [5–9], while others found no association between a higher BED and better local control [10, 11]. This question is critical in the context of the well-documented risk of subjecting large volumes of liver parenchyma to high-radiation doses, especially when treating large tumors [11, 12]. Acknowledging the uncertainty regarding dose response, the recently published American Society for Radiation Oncology (ASTRO) recommendations for liver cancer encompass a wide range of dose- fractionation regimens, taking into account tumor size and patient’s liver function [13].

At our institution, HCC lesions with a diameter less than or equal to 5 cm are typically treated with five-fraction stereotactic body radiotherapy (SBRT) to a BED_10_ of 100 Gy. This has been driven by the established high rate of local control and low toxicity of SBRT in this population [14]. Tumors with a larger diameter are treated with a more fractionated regimen, sometimes compromising on the radiation dose to meet normal tissue constraints. This heterogeneity in dose and fractionation schedules creates an opportunity to investigate the dose-response relationship of radiotherapy for local control and liver toxicity in small and large HCC tumors treated at our department. We hypothesized that higher radiotherapy doses would be associated with improved local control and similar toxicity compared to lower doses.

## Methods

### Data source and patient cohort

Patients with non-metastatic HCC treated at the University of Pennsylvania with radiotherapy between 2009 and 2018 were identified from ARIA patient information system (Varian Medical Systems, Palo Alto, CA). Clinical data were extracted from the hospital’s Epic electronic medical record (Epic Systems Corporation, Verona, WI) following approval of the institutional review board. Patients treated with any fractionation were included as long as the target received a BED_10_ dose of at least 60 Gy. BED was calculated based on the prescribed dose per fraction, the number of fractions delivered, and a presumed α/β = 10 in accordance with the recently published ASTRO clinical practice guidelines for liver cancer [13] and as mostly done by previous investigators [4, 15–18]. However, Given some uncertainty in the true α/β ratio, we repeated the analysis with BEDs calculated with α/β = 7 and α/β = 3. A BED_10_ threshold of 60 Gy was chosen as it equals an equivalent dose in 2 Gy fractions (EQD2) of 50 Gy, which, based on our institutional practice, would exclude patients treated with palliative intent (as opposed to definitive therapy for durable control). Of note, patients at our institution with Child-Pugh B 8–9 or C liver disease routinely undergo a treatment break to identify those who develop early radiation-induced liver function decline. The initial course of radiotherapy in these split course regimens is at least 60 Gy BED_10_.

### Outcome variables

The primary outcome in this study was local recurrence, defined as the progression of the tumor in the irradiated field. Local recurrences were diagnosed based on follow-up imaging — most commonly dynamic contrast-enhanced MRI performed routinely every three months under the National Comprehensive Cancer Network (NCCN) guidelines [19]. Follow-up time was calculated from the end of the radiotherapy course. Patients without recurrence or death were censored at the last follow-up. Patients were divided into low BED_10_ and high BED_10_ groups with a cutoff at the median BED_10_.

ALBI score, an objective prognostic measure of liver function developed and externally validated in large international cohorts of patients with HCC, was calculated based on albumin and bilirubin values [20, 21]. Toxicity endpoints were the maximum ALBI index in the four months following the initiation of radiotherapy and clinical liver function deterioration at four months and one year following the initiation of radiotherapy. Toxicity was defined based on the radiation start date as treatment courses in our sample ranged from 3 to 33 fractions. Clinical liver function deterioration was defined as new or increased ascites, bleeding due to coagulopathy, hepatic encephalopathy, hepatic hydrothorax, or variceal hemorrhage, and was determined from the electronic health record (EHR). Covariates, including age, sex, vascular invasion, ECOG performance status, baseline Child-Pugh group, and history of prior local therapy, were also obtained from the EHR. Baseline ALBI was defined as the mean ALBI score over the six weeks preceding the start of radiotherapy. Radiotherapy treatment data were obtained from the ARIA. Gross tumor volume (GTV) was measured using the treatment planning system and converted to equivalent sphere diameter based on the sphere volume formula V = 4πR^3^/3.

### Statistical methods

Variables were compared using a two-tailed Student’s T-test for continuous variables and Chi-square for categorical variables. To identify parameters associated with local recurrence following radiation therapy, a univariate and multivariate Cox proportional hazard analysis was performed. Local recurrence-free survival and overall survival were calculated using the Kaplan-Meier method. Survival outcome differences were evaluated using the log-rank test [22]. A propensity score-matched analysis with a 1:1 ratio and a caliper of 0.2 was conducted to balance the baseline characteristics between high and low-BED groups in order to minimize their effect on the oncologic outcome. A logistic regression model was used to calculate the propensity score, which included the following variables: Child-Pugh score and Treatment modality (photon vs. proton radiation therapy). Statistical analysis was performed using SPSS version 28 (SPSS, Inc., Chicago, Illinois, USA) and R version 3.4.2 (R Foundation for Statistical Computing, Vienna, Austria). For all calculations, P values < 0.05 were considered statistically significant.

## Results

### Patient characteristics and radiation treatment

The analytic cohort included one hundred twenty-eight patients (Table 1). A majority were male, in good performance status, with Child-Pugh A and ALBI Grade 2 baseline liver function, and had received prior local therapy. A third of the patients underwent SBRT. A minority of patients underwent a planned treatment break, with the vast majority completing the planned treatment course. The median BED_10_ was 78 Gy. The median follow-up time for the cohort was 16 months (484 days).

**Table 1:** Patient characteristics and treatment of the entire cohort

		All patients N = 128 (%)
Age (mean ± SD, years)		64.29 ± 9.19
Gender male		105 (82.0)
ECOG	0	63 (50.0)
	1	51 (40.5)
	2	11 (8.7)
	3	1 (0.8)
Child-Pugh Group	A	81 (66.4)
	B	34 (27.9)
	C	7 (5.7)
ALBI grade	1	23 (25.0)
	2	51 (55.4)
	3	18 (19.6)
Received prior local therapy		94 (73.4)
Vascular invasion		41 (32)
Dose per fraction	Conventional (180–200 cGy/Fx)	7 (5.5)
	Hypofractionated (201–500 cGy/Fx)	79 (61.7)
	Ultrahypofractionated (501 cGy/Fx≤)	42 (32.8)
Number of fractions	1–5	42 (32.8)
	6–20	37 (28.9)
	> 20	49 (38.3)
Planned treatment break		34 (26.6)
RT treatment modality	Proton	36 (28.1)
GTV volume (median, Q1, Q3 cm³)		113, 53, 282
GTV equivalent sphere diameter (median, Q1, Q3 cm)		6.0, 4.6, 8.1
BED_10_ (median, Q1, Q3, Gy)		78, 69, 98
Survival (median, months)		18.31

### Treatment outcome and predictors of local recurrence for the entire cohort

Of the 128 patients included in the study, 20 (15.6%) had a local recurrence in the irradiated field, and 71 (55.5%) died during the follow-up period. In patients with a local recurrence, the median time to recurrence was 197 days. Table 2 shows the results of a univariate and multivariate Cox proportional hazard analysis using the patient’s age, gender, ECOG performance status, Child-Pugh Group, ALBI grade, history of prior local therapy, vascular invasion, planned treatment break, proton radiation, GTV diameter, and BED_10_. Only BED_10_ over the median dose significantly predicted local recurrence-free survival. We next performed a multivariate Cox proportional hazard analysis for local recurrence-free survival. We included BED_10_ and GTV diameter, given previous studies demonstrating an association between tumor size and treatment outcomes in HCC [23, 24]. In our analysis, only BED_10_ was a significant predictor of local recurrence after radiation, with a hazard ratio of 0.324. No significant differences in the occurrence of local failure were noted between tumors with a diameter over and under 5 cm.

**Table 2:** Univariate and multivariate Cox regression analysis for local recurrence-free survival after radiation

		Number at risk	Cumulative probability of local recurrence %	Univariate analysis			P value	Multivariate analysis			P value
				HR	95.0% CI			HR	95.0% CI		
					Lower	Upper			Lower	Upper	
Age	> 70 years old	27	18.5	0.943	0.341	2.605	0.910				
Gender male		105	15.2	0.696	0.232	2.087	0.518				
ECOG	0	63	20.6	1	reference						
	1	51	11.8	0.779	0.296	2.055	0.614				
	2	11	9.1	0.491	0.064	3.760	0.494				
	3	1	0	0	0	-	0.988				
Child-Pugh Group	A	81	18.5	1	reference						
	B	34	11.8	0.984	0.322	3.005	0.977				
	C	7	14.3	1.422	0.186	10.885	0.734				
ALBI grade	1	23	17.4	1	reference						
	2	51	17.6	1.537	0.464	5.091	0.482				
	3	18	16.7	2.191	0.471	10.185	0.317				
Received prior local therapy		94	18.1	2.12	0.618	7.273	0.232				
Vascular invasion		128	32	1.081	0.451	2.821	0.873				
Planned treatment brake		34	11.8	0.762	0.254	2.283	0.627				
RT treatment modality	Proton vs. photon	36	8.3	0.446	0.131	1.524	0.198				
GTV diameter	> 5 cm vs. ≤ 5 cm	78	16.7	1.388	0.552	3.487	0.485	0.883	0.326	2.393	0.807
BED_10_	> 78 Gy vs. ≤ 78 Gy	59	8.5	0.341	0.124	0.942	0.038	0.324	0.108	0.972	0.044

Abbreviations: BED, Biologically Effective Dose; GTV, Gross tumor volume; ECOG, Eastern Cooperative Oncology Group; ALBI, Albumin-bilirubin prognostic score

### Impact of the radiation dose on local control in large and small tumors

**Fig. 1:**
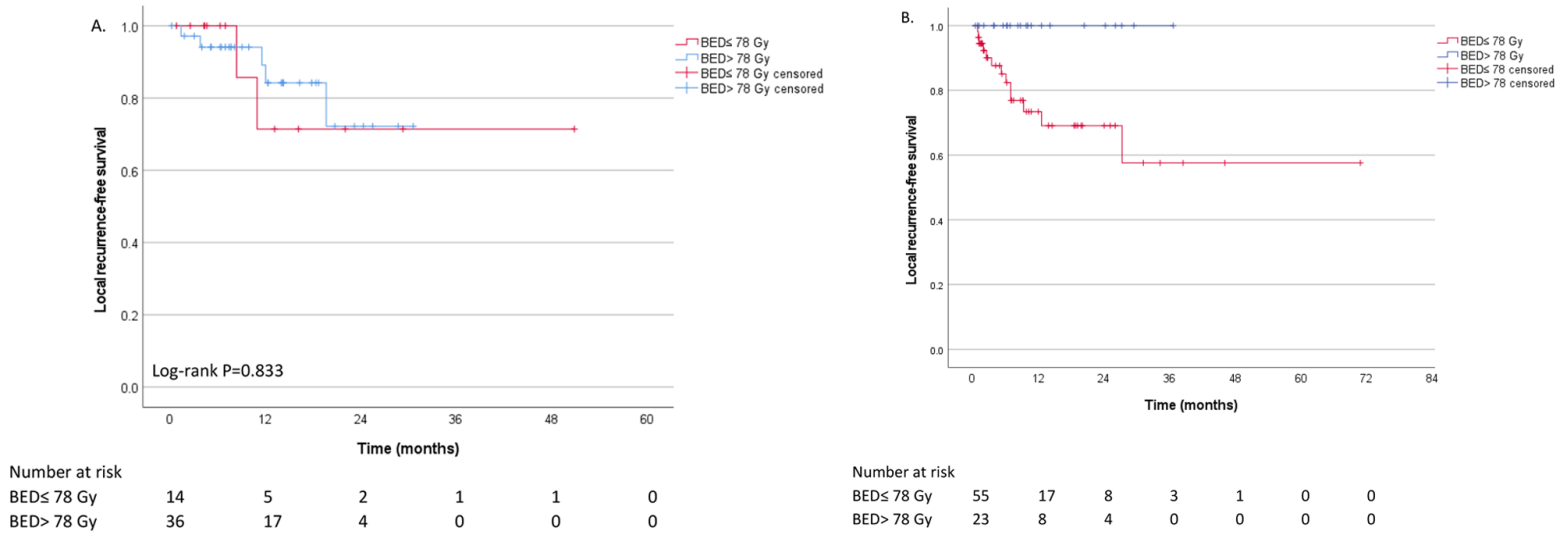
Subgroup analysis of local recurrence-free survival in HCC patients treated with BED over and under the median dose (calculated for α/β= 10 Gy). (**A**) GTV equivalent diameter≤ 5 cm (**B**) GTV equivalent diameter> 5 cm

Patients with small tumors (< 5 cm equivalent sphere diameter) had comparable mean age, Child-Pugh Group, and ECOG performance status compared to patients with large tumors (Table 3). Compared to patients with large tumors, patients with smaller tumors received significantly more ultrahypofractionated radiation regimens, higher BED, and fewer treated with proton therapy. There was no difference in local recurrence-free survival between patients with small and large tumors (Supplementary Fig. 1A); however, patients with large tumors had shorter overall survival (Supplementary Fig. 1B). We further explored the potential impact of radiation dose on local recurrence in small and large tumors. In tumors with a diameter under 5 cm, treating with a radiation dose higher than the median BED_10_ (78 Gy) did not result in improved local recurrence-free survival compared to a lower dose (Fig. 1A). However, in tumors with a diameter over 5 cm, treating with a higher than median BED_10_ significantly improved the local recurrence-free survival (Fig. 1B). Supplementary Fig. 2 shows the HR of each 10 Gy increase in BED_10_ as a function of tumor size. The plot shows that the impact of the 10 Gy increment in BED_10_ increased at a non-linear rate, with gradual improvement in the HR at a cutoff of 1.7 cm, with a steep benefit in tumors over 5 cm in diameter.

**Table 3:** Patient characteristics and treatment in small and large tumors

		GTV diameter N (%)		
		0–5 cm	5 cm<	P value
N (%)		50 (39.1)	78 (60.9)	
Age (mean ± SD, years)		63.61 ± 9.11	64.72 ± 9.27	0.506
Gender male		39 (78.0)	66 (84.6)	0.342
ECOG	0	26 (53.1)	37 (48.1)	0.460
	1	17 (34.7)	34 (44.2)	
	2	6 (12.2)	5 (6.5)	
	3	0 (0)	1 (1.3)	
Child-Pugh Group	A	31 (64.6)	50 (67.6)	0.271
	B	11 (22.9)	23 (31.3)	
	C	6 (12.5)	1 (1.4)	
ALBI grade	1	7 (19.0)	16 (28.6)	0.096
	2	18 (50.0)	33 (58.9)	
	3	11 (30.6)	7 (12.5)	
Received prior local therapy		38 (76.0)	56 (71.8)	0.599
Dose per fraction	Conventional (180–200 cGy/Fx)	3 (6.0)	4 (5.1)	< 0.001
	Hypofractionated (201–500 cGy/Fx)	11 (22.0)	68 (87.2)	
	Ultrafractionated (501 cGy/Fx≤)	36 72.0	6 (7.7)	
Number of fractions	1–5	36 (72.0)	6 (7.7)	< 0.001
	6–20	8 (16.0)	29 (37.2)	
	> 20	6 (12.0)	43 (55.1)	
Planned treatment break		13 (26.0)	21 (26. 9)	0.908
RT treatment modality	Proton	4 (8.0)	32 (41.0)	< 0.001
GTV volume (median, Q1, Q3 cm³)		24, 13, 43	314, 173, 538	< 0.001
GTV equivalent sphere diameter (median, Q1, Q3 cm)		3.5, 2.9, 4.3	8.4, 6.9, 10.0	< 0.001
BED_10_ (median, Q1, Q3, Gy)		100, 95, 100	69, 69, 78	< 0.001
Follow-up (median, months)		16.3	14.8	0.394

Abbreviations: BED, Biologically Effective Dose; GTV, Gross tumor volume; ECOG, Eastern Cooperative Oncology Group; ALBI, Albumin-bilirubin prognostic score

The results of our analyses using an α/β of 7 and 3 to calculate the BED are presented in Supplementary Tables 1, 2, and 3 and Supplementary Fig. 3A, 3B, 4A, 4B, 5A, and 5B. There were no differences compared to our primary analyses using an α/β of 10. In addition, we performed a propensity score matching based on the covariates: Child-Pugh Score and treatment modality (proton vs. photon). As can be seen in Supplementary Tables 4, the propensity-matched cohort in the low-BED group was evenly matched with the high-BED cohort in terms of baseline liver function and treatment modality. After propensity-matching, there was no difference in survival between the high and low-BED-treated groups, but the improved local recurrence-free survival after high-BED radiation treatment persisted (Supplementary Figs. 6, 7). Subgroup analysis reaffirmed that high BED-radiation treatment improves local control in tumors with a diameter over 5 cm but not smaller tumors (Supplementary Fig. 8A, B).

### Effects of radiation dose on liver toxicity

Table 4 compares liver toxicity at different time points, as a function of the BED_10_, in the entire cohort and patients with small and large tumors. Comparing patients treated with high vs. low BED_10_, there were no significant differences in liver function before radiation, four months after, and one year after completion of therapy in the entire cohort and the sub-group of patients with tumors < 5 cm. In the subset of patients with larger tumors, patients with the worst ALBI score at baseline received a lower radiation dose. However, when comparing high vs. low BED_10_, there was no difference in the mean maximum ALBI score and liver decompensation at four months and one year after radiation. Our institution’s constraints for the organs at risk for a fractionated regimen (7000 cGy in 20 fractions) and SBRT (5000 cGy in 5 fractions) are provided in Supplementary Table 5A, B.

**Table 4:** Effects of radiation BED_10_ dose on liver toxicity in the entire cohort and subgroup analysis based on GTV equivalent diameter

	All patients			GTV diameter ≤ 5 cm			5 cm < GTV diameter		
	BED ≤ 78	BED > 78	P value	BED ≤ 78	BED > 78	P value	BED ≤ 78	BED > 78	P value
Mean baseline ALBI score	-1.98 ± 0.68	-2.00 ± 0.82	0.914	-1.99 ± 0.91	-1.71 ± 0.89	0.394	-1.97 ± 0.62	-2.39 ± 0.52	0.018
Mean maximum ALBI score at four months	-1.54 ± 1.07	-1.61 ± 1.07	0.751	-1.49 ± 1.45	-1.39 ± 1.12	0.794	-1.56 ± 0.97	-1.94 ± 0.93	0.123
Clinical liver decompensation at four months (%)	22.4	19.3	0.674	30.8	22.9	0.574	20.4	13.6	0.492
Clinical liver decompensation at one year (%)	40.3	29.8	0.224	38.5	31.4	0.646	40.7	27.3	0.270

Abbreviations: ALBI, Albumin-bilirubin prognostic score; BED, Biologically effective dose

## Discussion

Our study explored the dose-response relationship of radiotherapy for local control and liver toxicity in small and large HCC tumors treated at our department. We found that only the radiation BED was a significant factor for predicting local tumor control following radiation therapy. Higher BED and improved local control were significant for tumors over 1.7 cm and paramount for patients with tumors over 5 cm equivalent sphere diameter. Patients with small tumors did not seem to benefit from dose escalation. In addition, we observed similar liver toxicity among patients treated with high vs. low dose radiotherapy, as measured by ALBI and clinical liver function deterioration. Therefore, patients with non-metastatic HCC tumors over 5 cm in diameter may benefit from escalated doses of radiotherapy without increasing the risk of liver toxicity. At the same time, in small tumors, the local control was excellent, even if a high BED could not be achieved.

Historically, radiotherapy was considered ill-suited to treating liver tumors because of the organ’s limited radiotolerance [25]. With the advent of conformal therapy, image guidance, and motion management techniques, radiotherapy was incorporated into the American Association for the Study of Liver Disease’s national guidelines as a potential treatment for inoperable HCC [2]. The optimal dose and fractionation of radiotherapy remain a matter of debate, and prior studies are heterogeneous in their patient populations, study designs, and analytic approaches, precluding a clear conclusion. Our study’s association between high-BED radiotherapy and tumor control depends on the GTV diameter. Previous studies of Korean and American patients showed improved outcomes with higher radiotherapy BED, including objective response rate, local recurrence, progression-free survival, and overall survival [5–9]. However, other non-randomized studies found no association between higher BED doses and improved outcomes [10, 11]. These contradictory findings may be due to the non-linear relationship between GTV size and the improved local control in higher BED radiation. Our results suggest that an equivalent sphere diameter of around 5 cm is a critical threshold at which the relative impact of BED increases substantially as GTV volume rises.

An important clinical question is how to deliver higher radiation doses safely without an increase in liver toxicity. Prior work using older, less conformal radiotherapy techniques showed more significant toxicity with higher doses of radiotherapy. Some groups have delivered escalated doses based on Normal Tissue Complication Probability models [26–28]. To avoid liver toxicity, at our institution, we prescribe a split-course regimen for patients with a Child-Pugh score greater than 7. Treatment is discontinued in patients who show early liver toxicity at one-month follow-up [29]. Additionally, we use more conservative mean liver dose constraints for patients with worse liver function: 28 Gy for Child-Pugh A 5–6 to 24 Gy for Child-Pugh B > 7. Reassuringly, we did not observe increased ALBI scores or clinical liver function deterioration in patients treated with higher doses of radiotherapy.

Our study is subject to potential limitations common to all retrospective analyses, primarily the existence of confounding factors. However, different from typical retrospective reviews, where heterogeneity of treatment might be a problem, herein, the heterogeneity of dose is an advantage in that it allows the observation of a dose response if one were to exist. We recognize that our median follow-up time may seem short. However, we would submit that the population we see in our clinic is advanced; in fact, 55% of patients have died within this follow-up interval. Hence, this is a mature series despite a relatively short follow-up time. We also recognize that avoidance of liver toxicity at higher radiation doses likely depends on an approach (involving split-course and differential liver constraints that varies with liver function) that is not widely practiced. In addition, although radiation-induced liver toxicity typically manifests shortly after radiation, commonly within three months [26, 30–33], there are a few reports of liver toxicity occurring later [30]. Given the natural history of cirrhosis, characterized by decompensation secondary to many various causes, the relationship to radiation is difficult to establish with any certainty after six months. Nevertheless, we recognize that a small number of late radiation-induced liver toxicities may have been missed, leading to a small underestimation of liver toxicity.

In conclusion, higher BED is associated with improved local control in patients with tumors larger than 5 cm in diameter. Patients treated with a higher dose do not appear to have a higher rate of liver toxicity. Hence, for these larger tumors, radiation oncologists should strive to deliver the highest possible dose that can be safely delivered, respecting organs-at-risk (OAR) tolerance. For tumors smaller than 5 cm in diameter, OAR tolerance should be prioritized, as compromising target coverage is not likely to result in a substantial loss of local control.

### Electronic supplementary material

Below is the link to the electronic supplementary material.

Supplementary Material 1

Supplementary Material 2

Supplementary Material 3

Supplementary Material 4

Supplementary Material 5

Supplementary Material 6

Supplementary Material 7

Supplementary Material 8

Supplementary Material 9

Supplementary Material 10

Supplementary Material 11

Supplementary Material 12

Supplementary Material 13

Supplementary Material 14

Supplementary Material 15

Supplementary Material 16

Supplementary Material 17

Supplementary Material 18

## Data Availability

Please contact the author for data requests. Data sharing: Data are not available for reuse.

## Abbreviations

ALBI: Albumin-bilirubin prognostic score
ASTRO: American Society for Radiation Oncology
BED: Biologically effective dose
ECOG: Eastern Cooperative Oncology Group
EHR: Electronic health record.
GTV: Gross tumor volume
HCC: Hepatocellular carcinoma
NCCN: National Comprehensive Cancer Network
OAR: Organs-at-risk
