# Supplementary material for: Radiation dose is associated with improved local control for large, but not small, hepatocellular carcinomas
Source: Radiat Oncol. 2023 Aug 11;18:133. doi: 10.1186/s13014-023-02318-0 (PMC10422771; doi:10.1186/s13014-023-02318-0)
Supplement: Supplementary file 1 — Supplementary Material 1 [file 13014_2023_2318_MOESM1_ESM.docx]

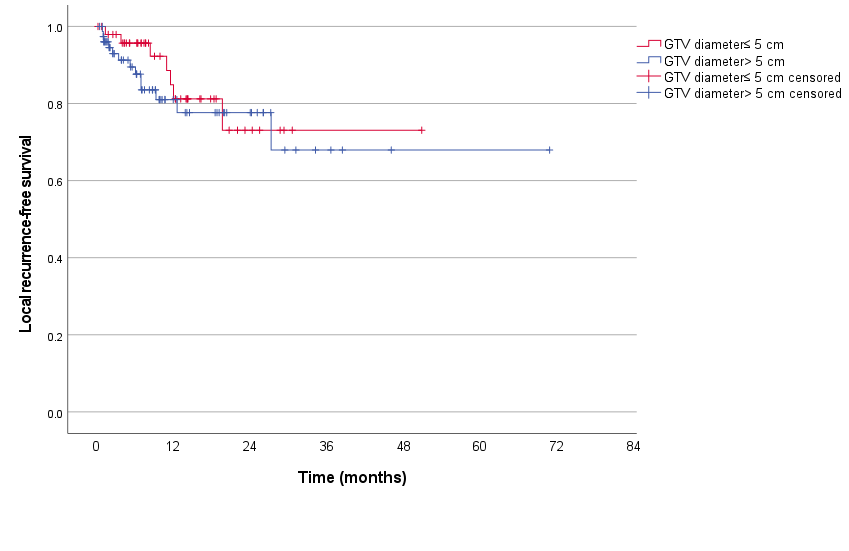
Supplementary Figure 1. Patterns of failure in HCC patients with tumors with a diameter over and under 5 cm. A. Local recurrence-free survival. B. Overall survival.

A.

| Number at risk |  |  |  |  |  |  |  |
| --- | --- | --- | --- | --- | --- | --- | --- |
| GTV diameter≤ 5 cm | 50 | 22 | 6 | 1 | 1 | 0 | 0 |
| GTV diameter> 5 cm | 78 | 25 | 12 | 4 | 1 | 1 | 0 |

Log-rank P=0.483
