# Supplementary figures and images for: Radiation dose is associated with improved local control for large, but not small, hepatocellular carcinomas
Source: Radiat Oncol. 2023 Aug 11;18:133. doi: 10.1186/s13014-023-02318-0 (PMC10422771; doi:10.1186/s13014-023-02318-0)

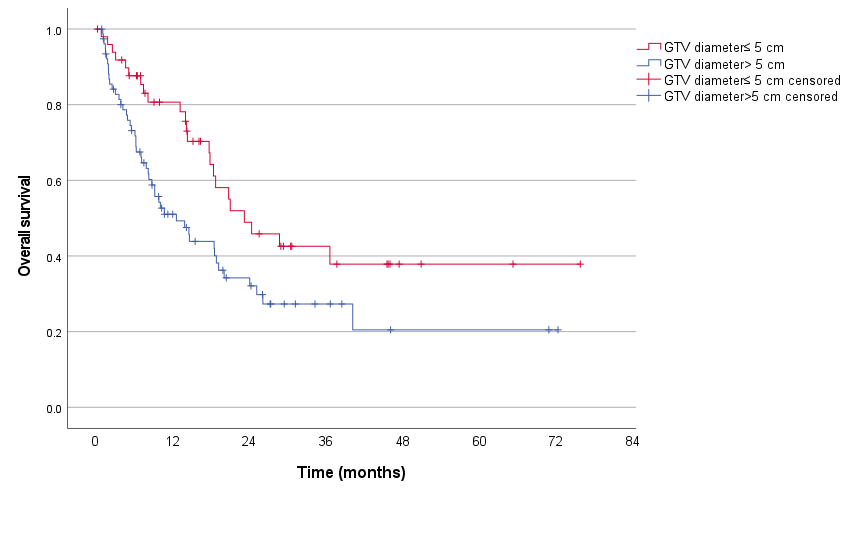


| Number at risk |  |  |  |  |  |  |  |  |
| --- | --- | --- | --- | --- | --- | --- | --- | --- |
| GTV diameter≤ 5 cm | 50 | 32 | 16 | 9 | 3 | 2 | 1 | 0 |
| GTV diameter> 5 cm | 78 | 29 | 16 | 6 | 2 | 2 | 0 | 0 |

Log-rank P=0.016

B.

Supplement: Supplementary file 2 — Supplementary Material 2 [file 13014_2023_2318_MOESM2_ESM.docx]

B.


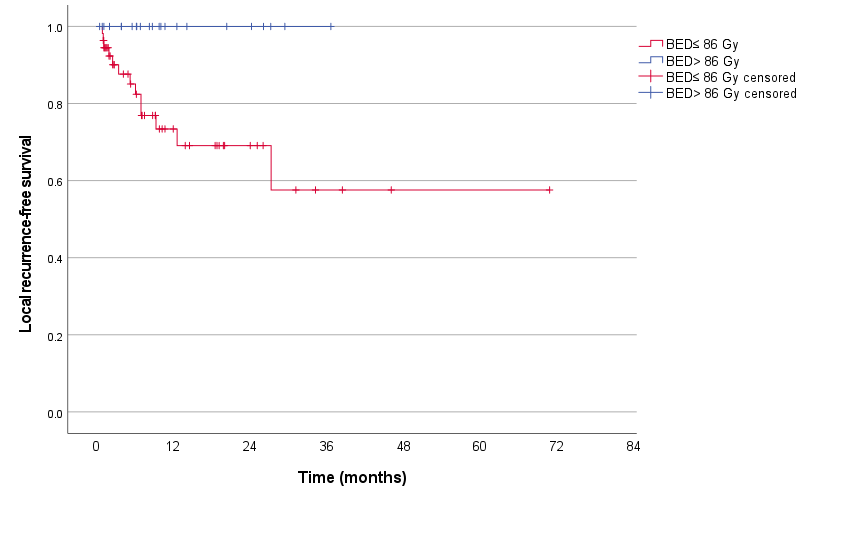


| Number at risk |  |  |  |  |  |  |  |
| --- | --- | --- | --- | --- | --- | --- | --- |
| BED≤ 86 Gy | 55 | 17 | 8 | 3 | 1 | 0 | 0 |
| BED> 86 Gy | 23 | 8 | 4 | 0 | 0 | 0 | 0 |

Log-rank P=0.017

Supplement: Supplementary file 5 — Supplementary Material 5 [file 13014_2023_2318_MOESM5_ESM.docx]

B.

| Number at risk |  |  |  |  |  |  |  |
| --- | --- | --- | --- | --- | --- | --- | --- |
| BED≤ 126 Gy | 56 | 17 | 8 | 3 | 1 | 0 | 0 |
| BED> 126 Gy | 22 | 9 | 6 | 1 | 0 | 0 | 0 |


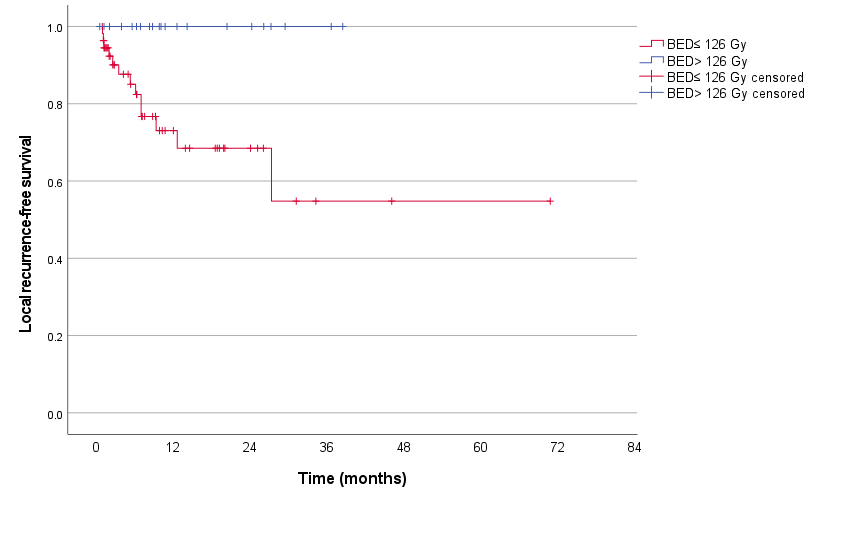


Log-rank P=0.012

Supplement: Supplementary file 7 — Supplementary Material 7 [file 13014_2023_2318_MOESM7_ESM.docx]

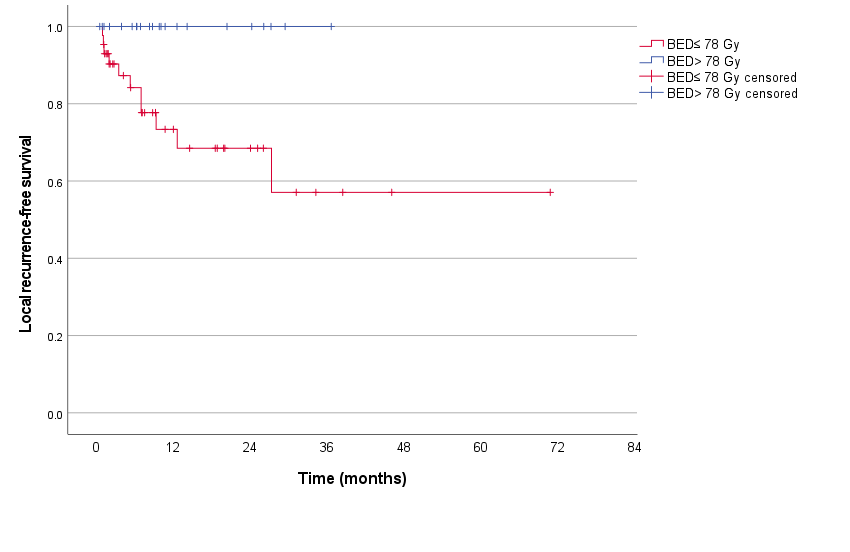


| Number at risk |  |  |  |  |  |  |  |
| --- | --- | --- | --- | --- | --- | --- | --- |
| BED≤ 78 Gy | 43 | 17 | 10 | 4 | 2 | 1 | 0 |
| BED> 78 Gy | 23 | 9 | 6 | 2 | 0 | 0 | 0 |

B.

Log-rank P=0.016

Supplement: Supplementary file 13 — Supplementary Material 13 [file 13014_2023_2318_MOESM13_ESM.docx]
