# Supplementary material for: Radiation dose is associated with improved local control for large, but not small, hepatocellular carcinomas
Source: Radiat Oncol. 2023 Aug 11;18:133. doi: 10.1186/s13014-023-02318-0 (PMC10422771; doi:10.1186/s13014-023-02318-0)
Supplement: Supplementary file 3 — Supplementary Material 3 [file 13014_2023_2318_MOESM3_ESM.docx]

Supplementary Figure 2. The hazard ratio of local failure per 10 Gy increases in BED_10_ as a function of tumor volume and diameter. X-axis downward ticks are deciles of GTV volume and the corresponding equivalent sphere diameter. Upward ticks represent each patient. The X-axis scale is logarithmic, but ticks are un-transformed volumes and diameters.


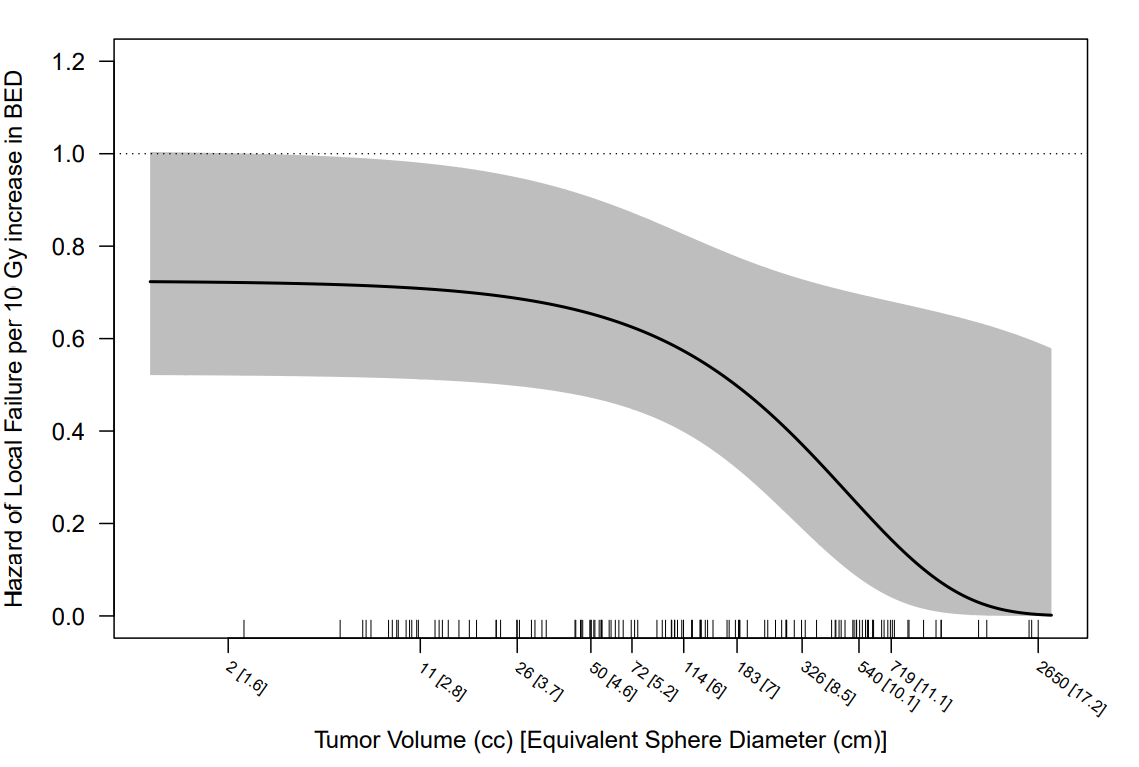


Abbreviations: BED, Biologically effective dose; cc, cubic centimeters; cm, centimeters.
