# Supplementary material for: Radiation dose is associated with improved local control for large, but not small, hepatocellular carcinomas
Source: Radiat Oncol. 2023 Aug 11;18:133. doi: 10.1186/s13014-023-02318-0 (PMC10422771; doi:10.1186/s13014-023-02318-0)
Supplement: Supplementary file 4 — Supplementary Material 4 [file 13014_2023_2318_MOESM4_ESM.docx]

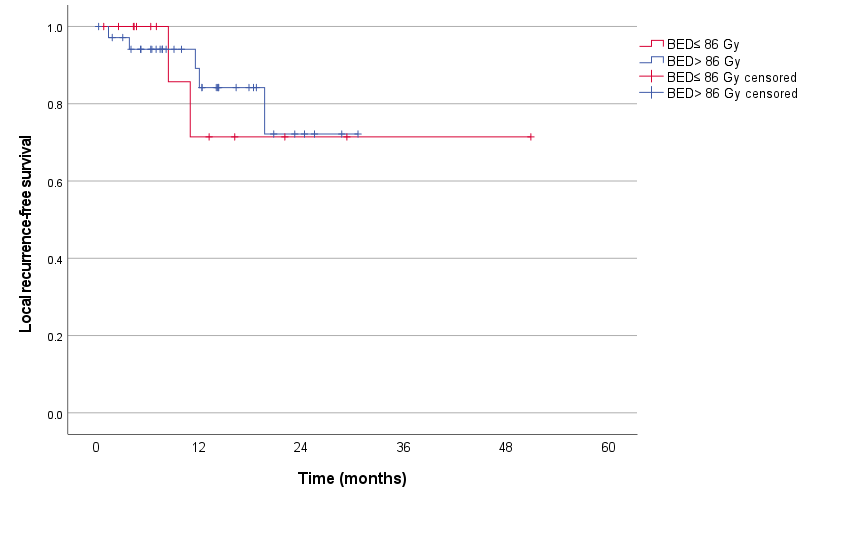
Supplementary Figure 3. Subgroup analysis of local recurrence-free survival in HCC patients treated with BED over and under the median dose 86 Gy (calculated for α/β= 7 Gy). A. GTV equivalent diameter≤ 5 cm B. GTV equivalent diameter> 5 cm.

A.

| Number at risk |  |  |  |  |  |  |
| --- | --- | --- | --- | --- | --- | --- |
| BED≤ 86 Gy | 14 | 5 | 2 | 1 | 1 | 0 |
| BED> 86 Gy | 36 | 17 | 4 | 0 | 0 | 0 |

Log-rank P=0.833
