# Supplementary material for: Radiation dose is associated with improved local control for large, but not small, hepatocellular carcinomas
Source: Radiat Oncol. 2023 Aug 11;18:133. doi: 10.1186/s13014-023-02318-0 (PMC10422771; doi:10.1186/s13014-023-02318-0)
Supplement: Supplementary file 10 — Supplementary Material 10 [file 13014_2023_2318_MOESM10_ESM.docx]

Supplementary Figure 6. Overall survival in propensity-matched HCC patients cohort treated with BED over and under the median dose 78 Gy (calculated for α/β= 10 Gy)

| Number at risk |  |  |  |  |  |  |  |  |
| --- | --- | --- | --- | --- | --- | --- | --- | --- |
| BED≤ 78 Gy | 57 | 31 | 18 | 11 | 7 | 5 | 3 | 0 |
| BED> 78 Gy | 57 | 30 | 15 | 6 | 2 | 0 | 0 | 0 |


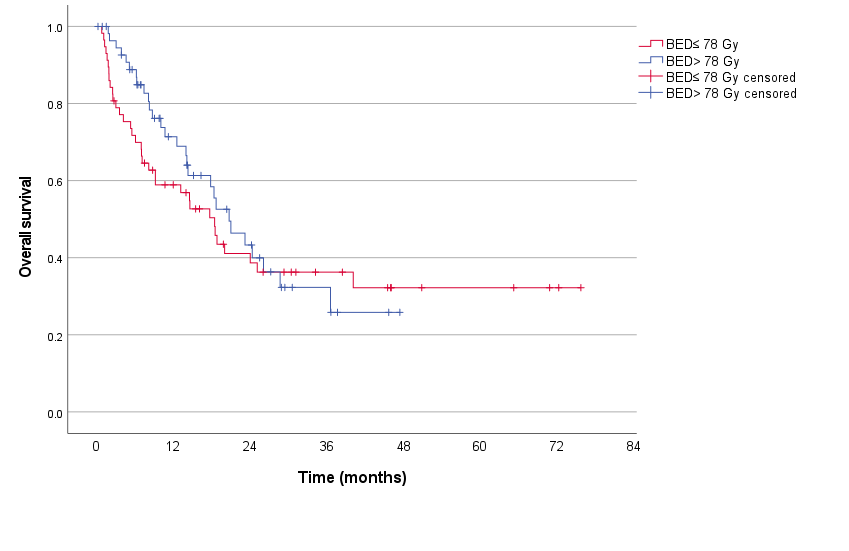


Log-rank P=0.519
