# Supplementary material for: Radiation dose is associated with improved local control for large, but not small, hepatocellular carcinomas
Source: Radiat Oncol. 2023 Aug 11;18:133. doi: 10.1186/s13014-023-02318-0 (PMC10422771; doi:10.1186/s13014-023-02318-0)
Supplement: Supplementary file 14 — Supplementary Material 14 [file 13014_2023_2318_MOESM14_ESM.docx]

Supplementary Table 1. Univariate and multivariate Cox regression analysis for local recurrence-free survival after radiation for BED for α/β=7
﷟

|  |  | Number at risk | Cumulative probability of local recurrence % | Univariate analysis | | |  | Multivariate analysis* | | |  |
| --- | --- | --- | --- | --- | --- | --- | --- | --- | --- | --- | --- |
|  |  |  |  | HR | 95.0% CI | | P value | HR | 95.0% CI | | P value |
|  |  |  |  |  | Lower | Upper |  |  | Lower | Upper |  |
| BED_7_ | >86 Gy vs ≤ 86 Gy | 59 | 8.5 | 0.341 | 0.124 | 0.942 | 0.038 | 0.324 | 0.108 | 0.972 | 0.044 |

Abbreviations: BED, Biologically Effective Dose; GTV, Gross tumor volume.

*Model adjusted for GTV diameter (> 5 cm vs ≤ 5 cm)
