# Supplementary material for: Radiation dose is associated with improved local control for large, but not small, hepatocellular carcinomas
Source: Radiat Oncol. 2023 Aug 11;18:133. doi: 10.1186/s13014-023-02318-0 (PMC10422771; doi:10.1186/s13014-023-02318-0)
Supplement: Supplementary file 17 — Supplementary Material 17 [file 13014_2023_2318_MOESM17_ESM.docx]

|  |  | Propensity matched cohort N= 114 (%) | | P value |
| --- | --- | --- | --- | --- |
|  |  | BED_10_≤ 78 Gy N=57 | BED_10_> 78 Gy N=57 |  |
| Age (mean± SD, years) |  | 62.01± 8.73 | 66.09± 9.20 | 0.170 |
| Gender male |  | 44 (72.6) | 50 (87.7) | 0.140 |
| ECOG | 0 | 28 (49.1) | 28 (50.9) | 0.130 |
|  | 1 | 26 (45.6) | 19 (34.5) |  |
|  | 2 | 2 (3.5) | 8 (14.5) |  |
|  | 3 | 1 (1.8) | 0 (0) |  |
| Child-Pugh Group | A | 34 (59.6) | 41 (71.9) | 0.247 |
|  | B | 20 (35.1) | 12 (21.1) |  |
|  | C | 3 (5.3) | 4 (7) |  |
| ALBI grade | 1 | 11 (26.2) | 10 (23.8) | 0.966 |
|  | 2 | 23 (54.8) | 24 (57.1) |  |
|  | 3 | 8 (18) | 8 (18) |  |
| Received prior local therapy |  | 43 (75.4) | 46 (80.7) | 0.497 |
| Vascular invasion |  | 19 (33.3) | 16 (28.1) | 0.542 |
| No planned treatment brake |  | 18 (31.6) | 14 (24.6) | 0.404 |
| RT treatment modality | Proton | 17 (29.8) | 15 (26.3) | 0.677 |
| GTV diameter | > 5 cm vs ≤ 5 cm | 43 (75.4) | 23 (40.4) | 0.001> |

Supplementary Table 4. Patient characteristics and treatment of the propensity-matched cohort
