# Supplementary material for: Radiation dose is associated with improved local control for large, but not small, hepatocellular carcinomas
Source: Radiat Oncol. 2023 Aug 11;18:133. doi: 10.1186/s13014-023-02318-0 (PMC10422771; doi:10.1186/s13014-023-02318-0)
Supplement: Supplementary file 18 — Supplementary Material 18 [file 13014_2023_2318_MOESM18_ESM.docx]

Supplementary Table 5. Constraints used in our institution’s protocol for organ at-risk constraints for HCC liver radiation. A. Fractionated regimen (7000 cGy in 20 fractions).B. Stereotactic body-radiotherapy regimen (5000 cGy in 5 fractions).

A.

| **Structure Name** | **DVH Objective** | **Evaluator** |
| --- | --- | --- |
| SpinalCord | D0.03cc[cGy] | <3500 |
| Duodenum | D0.03cc[cGy] | <4500 |
| Duodenum | D5%[cGy] | <4200 |
| Kidneys | D50%[cGy] | <1800 |
| *Liver-GTV | Mean[cGy] | <2400 |
| Stomach | D0.03cc[cGy] | <4400 |
| Stomach | D5%[cGy] | <4200 |
| Chestwall | D30cc[cGy] | <5400 |
| Chestwall | D10cc[cGy] | <6000 |
| AbdominalWall | D30cc[cGy] | <5400 |
| AbdominalWall | D10cc[cGy] | <6000 |
| Bowel_Large | D0.03cc[cGy] | <4500 |
| Bowel_Large | V4200cGy[cc] | <5 |
| Bowel_Small | D0.03cc[cGy] | <4500 |
| Bowel_Small | V4200cGy[cc] | <5 |
| Esophagus | D0.03cc[cGy] | <4500 |
| Heart | D0.03cc[cGy] | <4500 |
| Lungs | V2000cGy[%] | <20 |
| Lung_R | V2000cGy[%] | <20 |
| Lung_L | V2000cGy[%] | <20 |

* For Child-Pugh A, we accept variation< 2800 cGy

B.

| **Structure Name** | **DVH Objective** | **Evaluator** |
| --- | --- | --- |
| SpinalCord | D0.03cc[cGy] | <3000 |
| SpinalCord | D0.1cc[cGy] | <2500 |
| SpinalCord | D0.35cc[cGy] | <2300 |
| SpinalCord | D1.2cc[cGy] | <1450 |
| Esophagus | D0.03cc[cGy] | <3500 |
| Esophagus | D5cc[cGy] | <1950 |
| Heart | D0.03cc[cGy] | <3800 |
| Heart | D15cc[cGy] | <3200 |
| Chestwall | D0.03cc[cGy] | <4300 |
| Chestwall | D30cc[cGy] | <3250 |
| Skin | D0.03cc[cGy] | <3950 |
| Skin | D10cc[cGy] | <3250 |
| Stomach | D0.03cc[cGy] | <3200 |
| Stomach | D10cc[cGy] | <1800 |
| Duodenum | D0.03cc[cGy] | <3200 |
| Duodenum | D0.1cc[cGy] | <3000 |
| Duodenum | D5cc[cGy] | <1800 |
| Duodenum | D20cc[cGy] | <1250 |
| Bowel_Small | D0.03cc[cGy] | <3500 |
| Bowel_Small | D5cc[cGy] | <1950 |
| Bowel_Large | D0.03cc[cGy] | <3800 |
| Bowel_Large | D20cc[cGy] | <2500 |
| Lungs | D1500cc[cGy] | <1250 |
| Lungs | D1000cc[cGy] | <1350 |
| Kidneys | D200cc[cGy] | <1750 |
| Liver-GTV | CV1500cGy[cc] | >700 |
